# A reflection on ‘Aggregation-induced emission enhancement of a *meso*-trifluoromethyl BODIPY *via* J-aggregation’: from fundamental discovery to versatile sensing platforms

**DOI:** 10.1039/d5sc90236c

**Published:** 2025-11-04

**Authors:** Pooja Sharma, Jean Bouffard, Youngmi Kim

**Affiliations:** a Department of Chemistry, Research Institute of Basic Sciences, Kyung Hee University Seoul 02447 Korea youngmi.kim@khu.ac.kr; b Department of Chemistry and Nanoscience, Ewha Womans University Seoul 03760 Korea bouffard@ewha.ac.kr; c Graduate Program in Innovative Biomaterials Convergence, Ewha Womans University Seoul 03760 Korea

## Abstract

The BODIPY family of dyes enjoys great popularity for its advantageous luminsecence characteristics in solution. By contrast, most BODIPY dyes are quenched in the solid state. In rare instances, BODIPY derivatives have been found to undergo aggregation-induced emission (AIE) behavior, leading to unexpectedly red-shifted bands. In 2014, S. Choi *et al.* demonstrated that the 1,3,5,7-tetramethyl BODIPY derivative that bears a trifluoromethyl group at the *meso* position formed emissive J-aggregates, and reported their photophysical and structural characterization (S. Choi, J. Bouffard and Y. Kim, *Chem. Sci.*, 2014, **5**, 751, https://doi.org/10.1039/c3sc52495g). This commentary surveys the applications of this family of J-aggregating BODIPY dyes, and new instances of BODIPY J-aggregates that have emerged since that publication.

## Abstract 2

In celebration of our 15th anniversary and some of our most popular articles, Jean Bouffard and Youngmi Kim reflect on developments in aggregation-induced emission, building on their study published in *Chemical Science* on this topic (*Chem. Sci.*, 2014, https://doi.org/10.1039/c3sc52495g).

## Graphical abstract

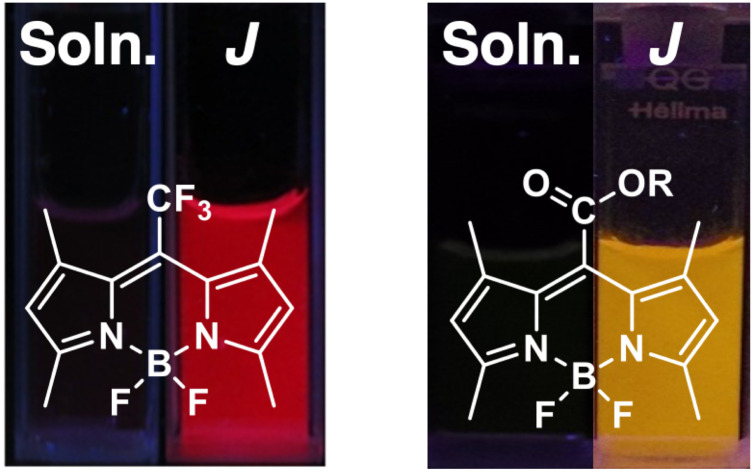

The first 4,4′-difluoro-4-bora-3*a*,4*a*-diaza-*s*-indacene (BODIPY) dye was reported in 1968.^1^ BODIPYs have since grown to become one of the most versatile classes of fluorophores because of their high molar absorption coefficients, robust thermal and photostabilities, high fluorescence quantum yields, and relatively straightforward and modular syntheses.^2^ Like most families of organic fluorophores, despite achieving high quantum yields (*Φ*_F_ > 0.5) in dilute solutions, BODIPYs have typically suffered from severe aggregation-caused quenching (ACQ), limiting their applications in condensed phases. Interestingly, it was observed early on that some BODIPY derivatives deviated from these trends, featuring characteristic broad and red-shifted emission bands under conditions of localized high concentrations. For example, BODIPY-tagged ceramides were found to undergo emission red shifts from *ca.* 515 to 620 nm at high mole fractions in lipid vesicles, enabling the selective fluorescence imaging of membranes, such as the Golgi apparatus, where bioaccumulation occurred.^3^ The red-emissive species was later identified as a ground-state dimer (or preassociated excimer).^4^ After the turn of the century, reports emerged of BODIPY dyes showing some features attributed to emissive J-aggregates.^5^ Dyes that J-aggregate show sharp bathochromically shifted absorption and emission bands that are nearly resonant, as first observed by Jelley and Scheibe in a pseudoisocyanine dye. According to exciton coupling theory, these result from packing structures that feature coplanar inclined transition dipoles with slip angles (*θ*) ≤ 54.7°. However, BODIPY J-aggregation remained equivocal due to the presence of mixed aggregated species, discordant features (*e.g.*, broad bands, significant Stokes shifts or quenched luminescence), or the lack of structural characterization in the solid state.^6^

In 2014, we reported the structural characterization of a *meso*-trifluoromethyl 1,3,5,7-tetramethyl BODIPY dye (CF_3_-BODIPY) that featured photophysical properties fully consistent with emissive exciton-coupled J-aggregates (Fig. 1, https://doi.org/10.1039/c3sc52495g).^7^

**Fig. 1:**
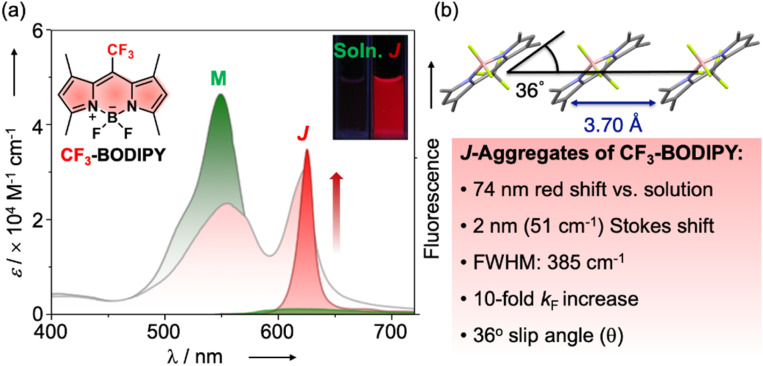
(a) Summary of spectroscopic and photophysical changes that accompany the formation of CF_3_-BODIPY J-aggregates (J, red curves) from the monomeric solvated dye (M, green curves). (b) Packing structure of CF_3_-BODIPY obtained from X-ray crystallography.

While most BODIPY dyes, exemplified by the *meso*-methyl BODIPY congener, exhibit severe fluorescence quenching in condensed phases, CF_3_-BODIPY demonstrated remarkable aggregation-induced emission (AIE) properties.^8^

In the solid state or suspended colloidal aggregates, CF_3_-BODIPY featured narrowed (FWHM = 385 cm^−1^) and red-shifted absorption and emission bands, minimal Stokes shift (51 cm^−1^), fluorescence rate constants 10-fold greater than the monomer in solution, and coplanar head-to-tail molecular arrangements with a small slip angle (*θ* = 36°) – characteristics entirely consistent with J-type aggregation according to exciton coupling theory.^5^ A spectral line shape analysis later established that CF_3_-BODIPY aggregates exhibited super-radiance with a spatial coherence number of *N* = 8.6 exciton-coupled chromophores.^9^ Rafal Klajn and co-workers later found that encapsulation of CF_3_-BODIPY within a Pd_6_^II^L_4_ coordination cage prevented the adoption of the intrinsic J-type packing structure found in its crystals or aggregates, the dyes instead associating therein to form H-type dimers.^10^

The discovery of emissive J-aggregation in CF_3_-BODIPY was significant not merely for the characterization of that one exceptional dye, but for confirming that, with the right molecular design, the predictable formation of BODIPY J-aggregates was within reach. Indeed, following this initial discovery, a systematic exploration of the structure–property relationships that govern J-aggregate formation in BODIPY dyes revealed that J-aggregation was not limited to CF_3_-BODIPY, but could be extended to *meso* ester-substituted derivatives of 1,3,5,7-tetramethyl BODIPYs.^11^ This work identified key design principles: the integration of (i) electron-withdrawing groups (EWG) at the *meso* position, coupled with (ii) sterically demanding flanking methyl groups at the 1,7-positions, which favored the formation of emissive J-aggregates (Scheme 1).

**Scheme 1:**
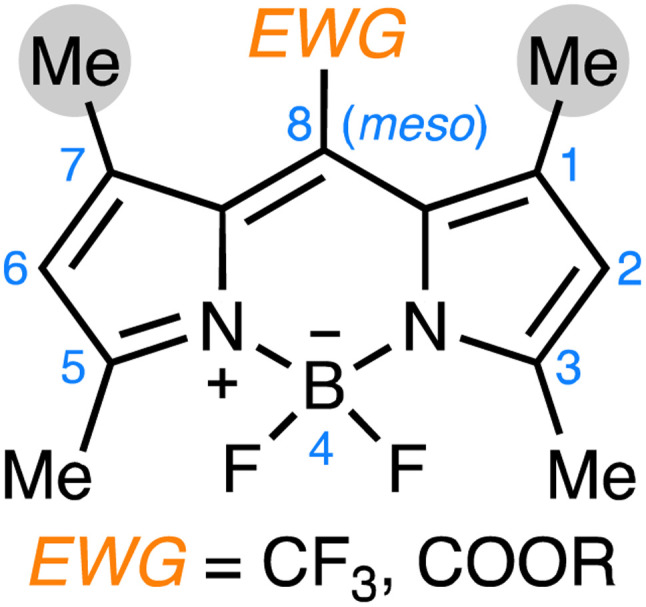
Common features among several J-aggregating BODIPY dyes: (i) electron-withdrawing groups at the *meso* position; (ii) flanking methyl groups at the 1,7-positions.

More importantly, unlike the rather inert *meso* trifluoromethyl substituent, the carboxyl group allowed for the preparation of functionalized derivatives that preserved emissive J-aggregate formation. Moreover, the reactivity of the ester group could be exploited to control either the formation or the destruction of these emissive aggregates. Transformations occurring at the carboxyl group that influenced interchromophore distance transduced it into a distinct optical signature. This property was exploited in the development of fluorogenic sensors and imaging probes for molecular recognition events.

Building on these fundamental insights, we designed several BODIPY J-aggregate-based fluorogenic sensing and imaging probes that harness control over their aggregation state, including the transformation of one J-aggregate into another (Fig. 2a), triggered J-aggregation of the monomers (Fig. 2b), or J-aggregate to monomer conversion (Fig. 2c).

**Fig. 2:**
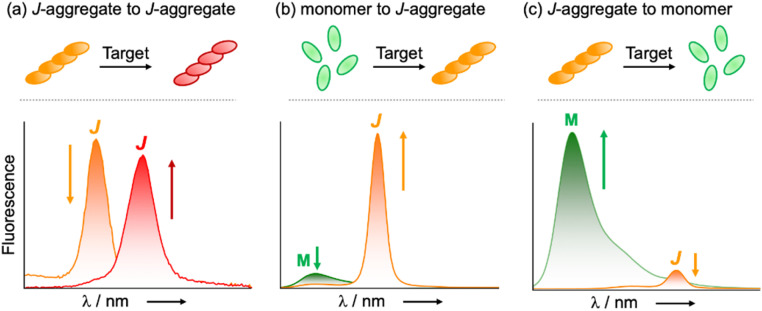
Examples of fluorogenic signal transduction schemes involving the formation of BODIPY J-aggregates that have been exploited in sensing applications since 2014.

A first foray into the design of sensory applications arose from the observation that the 2,6-dibromination of suspended orange-emissive J-aggregates of a *meso* ester 1,3,5,7-tetramethyl BODIPY dye gave red-emissive suspensions that preserved its J-aggregated structure. This phenomenon was exploited in the design of a probe for the selective detection of eosinophil peroxidase (EPO) activity. This enzyme converts hydrogen peroxide and bromide anions into the brominating agent HOBr, in contrast to myeloperoxidase (MPO), which instead generates HOCl from hydrogen peroxide and chloride ions.^12^ The resulting probe exploits a remarkable kinetic selectivity (≥1200 : 1) for HOBr-mediated bromination of the BODIPY over its HOCl-mediated chlorination. This J-aggregate to J-aggregate transformation (Fig. 2a) approach offered both high selectivity and high sensitivity (LOD: 0.09 ng mL^−1^ for EPO). Changhua Li and co-workers later presented evidence that J-aggregation does not merely tolerate halogenation at the 2,6-positions, but that halogen-bonding interactions in condensed states can sustain stacking arrangements that promote BODIPY J-aggregate formation.^13^

Triggered J-aggregation (Fig. 2b) can also serve as a powerful transduction mechanism for sensing in aqueous environments. In 2020, we reported that a pyridinium-tethered *meso* ester BODIPY dye formed J-aggregates in the presence of sulfated glycosaminoglycans.^14^ The monomer-to-J-aggregate transformation, driven by electrostatic interactions, resulted in the appearance of the narrow orange emissive band of the J-aggregated BODIPY dye. The probe was used to develop assays that distinguished the clinical anticoagulant heparin apart from closely related glycosaminoglycans: heparan sulfate, chondroitin sulfate, hyaluronic acid, and the adulterant oversulfated chondroitin sulfate.

BODIPY J-aggregation (Fig. 2b) was also exploited in the development of a fluorogenic probe for the detection of perfluorooctanesulfonate (PFOS) and perfluorooctanoic acid (PFOA).^15^ This probe featured a 1,3,5,7-tetramethyl BODIPY dye tethered through its *meso* ester to a hydrophobic decylimidazolium chain that formed green-emissive micelles of the monomeric dye in water. Upon binding one of the perfluoroalkyl substances (PFAS), spontaneous micelle disassembly and reorganization formed intensely orange-emissive J-aggregates within seconds. Because of its low cost, simple operation, rapid response, and high sensitivity (LOD = 0.18 ppb for PFOS), it was suggested as an on-site presumptive assay for the rapid localization of major PFAS point sources and their effluent plumes.

The reverse stimulus-induced conversion of BODIPY J-aggregates back to their monomeric state (Fig. 2c) and the dramatic optical changes that accompany disaggregation were also exploited for both therapeutic and diagnostic applications. In J-aggregated *meso* ester 1,3,5,7-tetramethyl BODIPY dyes, triggered ester hydrolysis through either physical, chemical, or biochemical means resulted in the formation of the *meso* carboxylate derivatives, which did not aggregate in water, and displayed the optical signatures of typical monomeric BODIPY dyes.

In a first application of triggered disaggregation (Fig. 2c), a *meso p*-azidobenzyl ester BODIPY dye was designed to spontaneously disaggregate upon light-activation.^16^ Under light irradiation, the azide photocage was reduced to the amine, which underwent a 1,6-elimination to yield the monomeric *meso* carboxylate BODIPY dye. The transformation is accompanied by a *ca.* 1250-fold luminescence increase of the green monomeric emission band at *ca.* 508 nm. Furthermore, this platform enabled the simultaneous uncaging of both the green-emissive dye and a bioactive molecule, such as the cancer drug chlorambucil, upon irradiation, providing real-time fluorescence monitoring of the release process in living cells.

In a second application of triggered disaggregation (Fig. 2c), a selective butyrate esterase probe was developed for the rapid identification of the pathogenic bacterium *Moraxella catarrhalis*.^17^ In aqueous buffer, the probe was resistant to nonspecific background hydrolysis. However, it underwent specific enzymatic cleavage, resulting in the disaggregation of orange-emissive J-aggregates to highly green-emissive monomeric carboxylates. The probe enabled the rapid (≤5 min) and accurate identification of *M. catarrhalis* for clinical diagnostics and microbiological analysis.

The stimulus-triggered disaggregation (Fig. 2c) of *meso* ester 1,3,5,7-tetramethyl BODIPY through ester hydrolysis and release of the green-emissive monomeric carboxylate was also exploited by other groups in diverse sensing applications. Changhua Li and co-workers also exploited *p*-substituted benzyl esters that undergo triggered hydrolysis through 1,6-elimination to develop probes that respond to reactive oxygen species (H_2_O_2_, ONO_2_^−^), hydrogen sulfide, or protease activity.^18,19^ Furthermore, diiodination of esters at the 2,6-positions did not hinder the J-aggregation of these dyes, enabling the development of stimuli-responsive photosensitizers for photodynamic therapy (PDT), where peroxynitrite-triggered disaggregation activated singlet oxygen generation for targeted cancer therapy.^19^ Weili Zhao and co-workers used the allyl *meso* ester of the J-aggregating 1,3,5,7-tetramethyl BODIPY to develop an assay for the detection of palladium.^20^ In this instance, a Pd-catalyzed Tsuji–Trost-type reaction resulted in the hydrolysis of the J-aggregated allyl ester to the green-emissive monomeric *meso* carboxylate BODIPY dye in solution, providing an intense fluorogenic response with fast kinetics.

Since the publication of the original *Chemical Science* article in 2014,^7^ additional fully characterized emissive BODIPY J-aggregates that possess congruent optical signatures (*i.e.*, significantly narrowed and red-shifted absorption and emission bands that are nearly resonant, and increased radiative rates), though not derived from the same 1,3,5,7-tetramethyl BODIPY platform, have also been reported.

In 2015, Zhijian Chen and co-workers reported an amphiphilic BODIPY derivative bearing quaternized propargylammonium groups at B in place of fluoride substituents.^21^ Though water-soluble, these dyes self-assembled at high concentrations through a nucleation-growth supramolecular polymerization mechanism. The resulting self-assembled vesicles combined the optical signatures of both H-type and J-type aggregation. The same group later reported BODIPY derivatives whose self-assembly in hydrocarbon solvents was driven by hydrogen bonding between ethynyluracil substituents appended at the 2,6-positions.^22^ The resulting J-aggregates featured coherence numbers as high as *N* ≈ 9, and thin films of these aggregated dyes were shown to reach high dichroic ratios through mechanical (rubbing) alignment techniques. Derivatives featuring bulkier alkyne substituents at B exhibited much greater quantum yields in the solid state than those with the more common BF_2_ group.

In 2017, the Daniel B. Werz lab reported the iterative synthesis of ethylene-bridged BODIPY oligomers through α–α oxidative couplings, up to the octamer.^23^ This synthetic tour-de-force has produced what may be the most impressive example of BODIPY J-aggregation to date, with a cryptopyrrole-derived hexamer featuring exceptionally narrowed absorption bands (286 cm^−1^), minimal Stokes shift (62 cm^−1^) and a quantum yield approaching unity in THF, where increased radiative rates outcompeted non-radiative deactivation processes such as internal conversion.^24^ Related features have sometimes been observed in methylene-bridged BODIPY dimers.^25^

Phenanthrene-fused BODIPYs that form J-aggregates with coherent domains of about 6 units in both water and hydrocarbon solvents were also reported by Lijuan Jiao, Erhong Hao, and co-workers in 2020.^26^ Though the aggregates were weakly luminescent, their bathochromic and hyperchromic absorption bands made them suitable photothermal therapy agents under 808 nm laser irradiation.

Finally, it should be noted that numerous instances of substantiated J-aggregation in the related family of aza-BODIPY dyes have also been reported.^27^

The discovery that 1,3,5,7-tetramethyl BODIPY dyes – first those bearing a *meso* trifluoromethyl group (CF_3_-BODIPY), then *meso* ester groups – demonstrably formed emissive exciton-coupled J-aggregates has provided spectroscopic, photophysical and structural guidelines that help distinguish them from other types of red-shifted aggregates, such as BODIPYs that instead form preassociated excimers in condensed phases. Furthermore, the J-aggregation behavior of these dyes was found to be highly predictable and reproducible, which enabled the design of fluorogenic probes, imaging agents and phototherapy agents. In view of their potential utility, it may be surprising that only a few BODIPY platforms aside from the 1,3,5,7-tetramethyl derivatives have been unambiguously shown to form genuine emissive J-aggregates. At this stage, the discovery of new J-aggregating dyes remains largely a serendipitous endeavor. Researchers should be reminded that a single molecular dye can form a multitude of self-assembled structures. Hence, due to aggregation pathway complexity, many commonly known or even commercially available fluorophores may later be revealed as overlooked J-aggregators. Until then, those await the fortuitous experimenter and opportune conditions that will coax them out of solution and into the right kind of kinetically trapped or thermodynamically stabilized self-assembled exciton-coupled structures.^28–30^

## Author contributions

Pooja Sharma, Jean Bouffard and Youngmi Kim wrote the manuscript.

## Conflicts of interest

The authors declare no competing interests.

## Data Availability

There is no additional data associated with this article.
